# An uncommon cause of diplopia: do not forget Brown syndrome

**DOI:** 10.1055/s-0044-1787798

**Published:** 2024-07-04

**Authors:** Caio César Diniz Disserol, Amanda Maieski, Samia Talise El Horr de Moraes, James Yared

**Affiliations:** 1 Universidade Federal do Paraná, Complexo do Hospital de Clínicas, Departamento de Neurologia, Curitiba PR, Brazil.; 2 CETAC Diagnóstico por Imagem, Neurorradiologia, Curitiba PR, Brazil.

**Figure 1:**
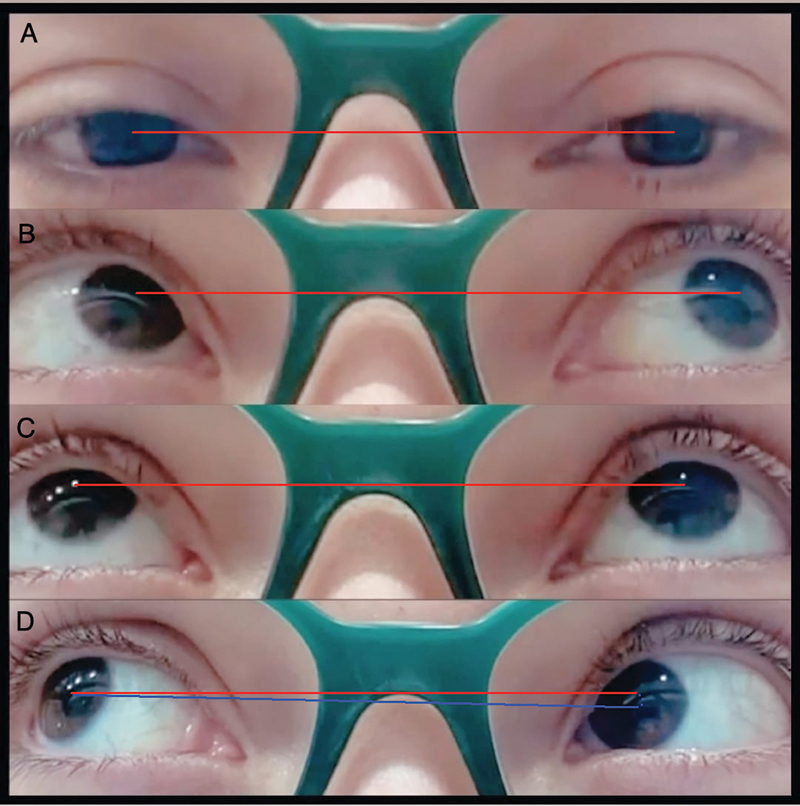
Horizontal red line highlighting normal primary gaze position ( **A** ), with supraversion in the left upgaze ( **B** ), and midline supraversion ( **C** ). Left hypotropia in the right upgaze, highlighted by the oblique blue line at the center of the pupils – the position that causes oblique diplopia in the patient ( **D** ).

**Figure 2:**
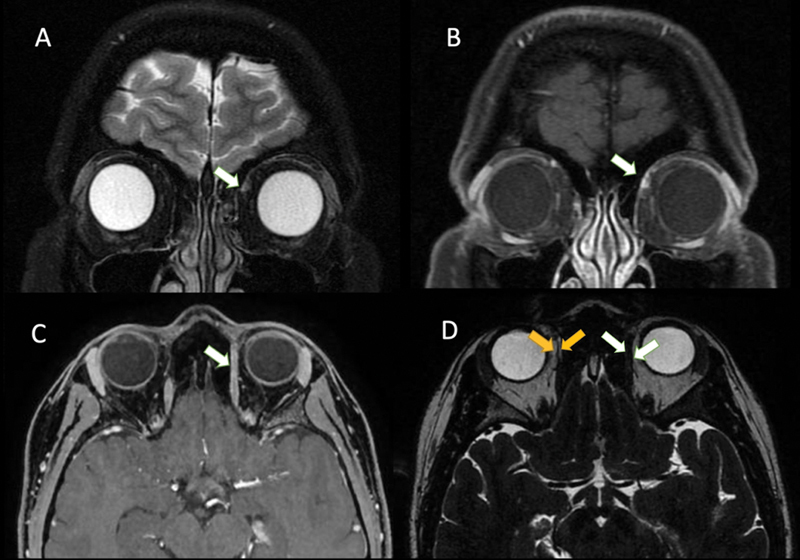
Orbital magnetic resonance imaging (MRI) scan. Left superior oblique muscle (SOM) tendon hyperintensity on coronal T2 ( **A** ). Postcontrast T1 with fat suppression showing left SOM tendon sheath gadolinium enhancement ( **B,C** ). Thickening of the left SOM tendon ( **white arrows** ) on axial T2 fast imaging employing steady-state acquisition (FIESTA) ( **D** ). The yellow arrow indicates normal thickness of the right SOM tendon.

A 30-year-old woman developed a new-onset orbital pain and vertical binocular diplopia in the right upgaze within 3 days. An examination revealed normal primary gaze position and left hypotropia in the right upgaze ( Figure 1 ), unreversed with forced duction. The pupils and left eye excycloduction were normal. A magnetic resonance imaging (MRI) scan revealed superior oblique muscle (SOM) tenosynovitis ( Figure 2 ). No infectious, autoimmune, metabolic or rheumatological etiologies were identified, and we concluded it was a case of idiopathic Brown syndrome (BS). The symptoms were resolved within one week of the administration of prednisone.

Contrary to inferior oblique muscle palsy, the limitation of supraduction in adduction in BS is unreversed with forced duction. 1 Brown syndrome is commonly congenital, with an onset in childhood. Acquired BS is idiopathic or due to surgery, trauma, tendon cysts, sinusitis, orbital tumors or rheumatological diseases. 2 The present report is to alert clinicians about this rare cause of diplopia for prompt diagnosis and treatment.
